# Health-related quality of life and demographic profiling of patients with selected noncommunicable diseases

**DOI:** 10.4102/hsag.v31i0.3379

**Published:** 2026-06-30

**Authors:** Lynn Smith, Heather Morris-Eyton

**Affiliations:** 1 Department of Sport and Movement Studies, Faculty of Health Sciences, University of Johannesburg, Johannesburg, South Africa

**Keywords:** noncommunicable diseases, health-related quality of life, patient-reported outcomes, chronic disease, multimorbidity

## Abstract

**Background:**

Health-related quality of life (HRQoL) reflects physical, mental and socioeconomic well-being. In people living with chronic noncommunicable diseases (NCDs), HRQoL is an important indicator of treatment effectiveness and long-term outcomes. Understanding domain-specific strengths and vulnerabilities can support person-centred care.

**Aim:**

To determine the demographic profile and HRQoL of adults living with hypertension, type 2 diabetes mellitus and/or cardiovascular disease (CVD).

**Setting:**

Public and private healthcare facilities in the Johannesburg metropolitan district, South Africa.

**Methods:**

A quantitative cross-sectional survey was conducted. Using purposive stratified random sampling, 257 adults with hypertension, type 2 diabetes mellitus and/or CVD completed the 37-item Smith Toolkit for Integrated Health-Related Quality of Life. The instrument assesses physical, mental and socioeconomic domains and demonstrated strong reliability. Data were collected in person and online and analysed descriptively using SPSS version 28.

**Results:**

Overall HRQoL was moderate to high (77.70 ± 12.91). Physical health scores were strong (80.31 ± 15.78), with favourable ratings for symptoms, work functioning and physical limitations, although vitality was lower (52.40 ± 34.01). Mental health scores were moderate (72.02 ± 17.19), with lower self-efficacy and evidence of psychological challenges. Socioeconomic health was strong (81.49 ± 17.66), supported by high perceived social support, financial stability and relationship quality.

**Conclusion:**

Participants generally reported good HRQoL despite living with chronic NCDs. However, lower vitality and self-efficacy indicate important gaps in psychological and behavioural functioning. Interventions targeting fatigue, resilience and self-management may further improve well-being and health outcomes.

**Contribution:**

This study provides context-specific evidence on HRQoL among South Africans living with NCDs and highlights the value of holistic, interdisciplinary and person-centred care.

## Introduction

Noncommunicable diseases (NCDs) remain a leading cause of morbidity and mortality globally, accounting for over half of all deaths in South Africa (World Health Organization [WHO] 2018, 2023). The global epidemiological transition has seen a steady rise in NCDs such as cardiovascular disease (CVD), hypertension and type 2 diabetes, which now dominate disease burden profiles previously characterised by infectious conditions (Mayosi et al. 2009; Wu et al. 2024). In low- and middle-income countries (LMICs), this rise has been particularly alarming due to constrained health systems, limited surveillance capacity and persistent socioeconomic inequities that heighten vulnerability (Barry et al. 2025). The coexistence of chronic and infectious diseases has generated a complex double burden, straining already overextended healthcare resources and limiting the sustainability of preventive interventions.

Globally, the burden of NCDs is driven by behavioural and environmental risk factors, including unhealthy diet, physical inactivity, tobacco use and harmful alcohol consumption (WHO 2023). ‘These determinants are amplified by rapid urbanisation and socioeconomic transitions that alter lifestyle behaviours and health-seeking practices’ (Luo & Wang 2022). In South Africa, NCDs account for the majority of premature deaths, particularly among working-age adults, leading to reduced productivity and increased economic strain on households and the national health system (Implementation Science for Non-Communicable Diseases 2025; Mayosi et al. 2009; WHO 2023). Furthermore, inequities in health literacy, access to care and adherence to long-term treatment perpetuate disparities in disease outcomes across income groups and geographic regions.

Cardiovascular diseases, diabetes and hypertension are closely interlinked. Diabetes increases cardiovascular mortality risk by 1.5–3 times compared to non-diabetic individuals (Dal Canto et al. 2019; Glovaci, Fan & Wong 2019; International Diabetes Federation 2023). In South Africa, 73% of patients with diabetes present with at least one cardiovascular comorbidity (Mutyambizi et al. 2017). Shared risk factors such as obesity, dyslipidaemia, endothelial dysfunction and chronic inflammation connect these diseases, amplifying cardiovascular risk (Petrie, Guzik & Touyz 2018). Early detection and integrated management of these risk factors are crucial to mitigating microvascular and macrovascular complications (Petrie et al. 2018). However, despite increasing disease prevalence, the healthcare response remains predominantly biomedical, often neglecting psychosocial and contextual determinants of health.

Recent shifts towards person-centred and holistic models of care emphasise that treatment success in chronic disease management extends beyond physiological control to encompass patient well-being and life satisfaction.

Health-related quality of life (HRQoL) has emerged as a critical indicator of overall well-being, capturing the multidimensional effects of illness and treatment on physical, mental and social functioning (Kangas et al. 2025; Witts et al. 2024). Health-related quality of life is recognised as a key predictor of mortality and health outcomes, with better HRQoL associated with lower risk of death and greater clinical stability (Phyo et al. 2020). Moreover, the assessment of HRQoL aligns with global efforts to strengthen universal health coverage through value-based and patient-centred healthcare delivery (WHO 2023).

Despite growing international emphasis on HRQoL in chronic disease care, there remains a paucity of empirical data from African settings, with comparatively few large population studies on multidimensional HRQoL outcomes in this context (Bakzaza et al. 2024; Witts et al. 2024). South Africa, characterised by cultural diversity and pronounced socioeconomic inequality, offers a unique context in which the determinants of HRQoL are deeply intertwined with access to care, social capital and community resilience. Evidence from South Africa illustrates that disparities in HRQoL exist between low-resourced urban and rural populations living with NCDs, highlighting how socioeconomic and environmental contexts shape well-being (Ramalivhana, Veldsman & Moss 2024). Understanding these dynamics is vital for designing equitable, context-sensitive interventions that improve long-term outcomes for people living with chronic conditions in African health systems.

Accordingly, this study aimed to determine the demographic profile and HRQoL of patients with hypertension, type 2 diabetes and/or CVD using the Smith Toolkit for Integrated Health-Related Quality of Life, a multidimensional instrument designed to capture physical, psychological and socioeconomic aspects of well-being.

## Research methods and design

### Research setting

Participants were recruited from a range of public and private healthcare facilities in the Johannesburg metropolitan district that provide services for patients living with hypertension, type 2 diabetes mellitus and CVD. Questionnaires were completed either onsite in a private area within the healthcare facility or remotely through a secure Google Forms link, depending on participant preference and accessibility.

### Study design

A quantitative, cross-sectional design was used. Descriptive and quantitative data were collected in Johannesburg, South Africa.

### Population and sample

Adults diagnosed with hypertension, type 2 diabetes mellitus and/or CVD receiving treatment at selected public and private healthcare facilities in South Africa were recruited using purposive stratified sampling. This approach ensured diversity in demographic and clinical characteristics relevant to the target population. Participants completed the questionnaire either onsite through hard copy questionnaires at the healthcare facility or remotely via a secure Google Forms link.

### Site of study and sampling

A sample of 257 patients with hypertension, type 2 diabetes mellitus and CVD or a combination of two or three of the conditions completed the questionnaire at various public and private healthcare facilities in Johannesburg, South Africa. A total of 10 healthcare facilities in the City of Johannesburg Metropolitan Municipality participated in the study, comprising four public and six private facilities. Private facilities were included to enhance diversity in socioeconomic status, healthcare access and patient experiences.

### Selection and recruitment of participants

Healthcare practitioners at these facilities were briefed on the study aims, inclusion criteria and ethical procedures. Practitioners identified eligible patients during routine consultations and invited them to participate.

The inclusion criteria were adults (≥ 18 years) with a confirmed diagnosis of one or more of the target conditions, and who attended either of the selected health facilities.

### Data collection and tool

The toolkit used in this study, the Smith Toolkit for Integrated Health-Related Quality of Life (STI-HRQoL), was developed as part of a larger research initiative and is not yet widely established in the literature (Smith & Morris-Eyton 2023). The STI-HRQoL comprises three domains, namely physical health, mental health and socioeconomic health, each consisting of distinct subscales. The questionnaire consists primarily of closed-ended items measured using a Likert-type response scale, allowing participants to indicate the extent to which they agreed with or experienced various health-related statements. The physical health domain includes recent physical limitations, symptoms, work-related functioning and vitality. The mental health domain assesses self-efficacy, challenges to mental health, coping behaviours and treatment literacy. The socioeconomic domain includes perceived social support, financial aspects and relationships. The questionnaire indicators, subscales and totals were calculated and recorded as percentages. In a range between 0% and 100%, the higher percentages reflected a better HRQoL.

After receiving an information sheet and providing written consent, questionnaires were administered.

Questionnaires were given to healthcare practitioners to administer to their patients who met the inclusion criteria to participate in this study. Completion of the questionnaires took place at the healthcare facility where participants were seeking treatment, and the researchers were available to provide clarification where necessary but did not influence participant responses. Upon request, the link on Google Forms was made available for remote access, particularly those with limited time, mobility restrictions or accessibility challenges.

### Data analysis

Quantitative data were captured on the Statistical Package for the Social Sciences (SPSS) version 28. Descriptive statistics were computed for all HRQoL domains. Sociodemographic variables included age, gender, ethnicity, education, employment and smoking status. The means, minimum and maximum ranges for age were calculated. The scores calculated included the HRQoL of the physical component, mental component, socioeconomic components and overall health and are presented as percentages.

### Ethical considerations

Ethical clearance to conduct this study was obtained from the Faculty of Health Sciences Research Ethics Committee of the University of Johannesburg (No. REC-01-04-2018). Ethical principles of autonomy, beneficence, non-maleficence, justice, privacy and confidentiality were maintained throughout the study. Participants were provided with an information letter and were required to provide consent to participate in the research. Participation was voluntary, and participants could withdraw at any stage without penalty. Questionnaires were completed in private settings or via a secure Google Forms link, and all data were pseudonymised and stored securely.

## Results

The results present the demographic profile and HRQoL outcomes of adults living with hypertension, type 2 diabetes mellitus and/or CVD. Descriptive statistics were used to summarise participant characteristics and HRQoL scores across the physical, mental and socioeconomic domains of the STI-HRQoL instrument.

### Respondent’s demographic characteristics

The demographic characteristics of the study sample, followed by the HRQoL outcomes across the physical, mental and socioeconomic domains of the STI-HRQoL instrument, are presented in Table 1.

**TABLE 1:** Demographics (*N* = 257).

Variable	Category	Frequency (*n*)	% distribution
Gender	Male	119	46.3
	Female	138	53.7
Ethnicity	Black African people	121	47.1
	Coloured people	49	19.1
	Indian and/or Asian people	22	8.6
	White people	65	25.3
Home language	English	111	43.2
	Afrikaans	45	17.6
	African language	118	45.9
Highest level of education	Did not finish high school	55	21.4
	Grade 12 or matric	111	43.2
	Certificate	12	4.7
	Diploma	40	15.6
	Degree	39	15.2
Type of employment	Permanent	118	45.9
	Temporary or contract	27	10.5
	Unemployed	61	23.7
	Retired	51	19.8
Smoking status	Never smoked	162	63
	Current smoker	66	25.7
	Former smoker	29	11.3
Diagnoses	Hypertension	175	68
	Type 2 diabetes	89	35
	Cardiovascular disease	60	23
Healthcare facility	Private	78	30
	Public	179	70

Note: Age (years): Mean = 51.85, Range = 21–88.

The sample demographics are outlined in Table 1. A total of 257 participants were included in the study, with a mean age of 51.85 years (range: 21–88 years). The sample comprised slightly more females (53.7%, *n* = 138) than males (46.3%, *n* = 119). Ethnic distribution was diverse, with 47.1% Black African, 25.3% White, 19.1% Coloured and 8.6% Indian and/or Asian participants. Regarding home language, 43.2% reported English, 17.6% Afrikaans and 45.9% an African language as their primary language.

Educational attainment varied: 21.4% of participants had not completed high school, 43.2% had completed Grade 12 or Matric, 4.7% held a certificate, 15.6% a diploma and 15.2% a university degree. Employment status indicated that 45.9% were permanently employed, 10.5% employed on a temporary or contract basis, 23.7% were unemployed and 19.8% were retired.

Lifestyle and health-related characteristics showed that the majority had never smoked (63%), while 25.7% were current smokers, and 11.3% were former smokers. The prevalence of chronic conditions was high, with 68% diagnosed with hypertension, 35% with type 2 diabetes and 23% with CVD. Most participants accessed healthcare through public facilities (70%), with the remainder attending private facilities (30%).

A total of 110 participants (42.8%) presented with comorbid NCDs, indicating a substantial burden of multimorbidity within the sample. Overall, the cohort represents a middle-aged, socioeconomically and ethnically diverse population with a high burden of chronic disease and varying levels of education and employment. These characteristics provide important context for interpreting intervention outcomes and highlight the relevance of tailored health strategies for populations with high multimorbidity and varied access to healthcare.

### Health-related quality of life of the sample

Table 2 presents the mean percentage scores and standard deviations for the physical, mental and socioeconomic HRQoL domains and subscales measured using the STI-HRQoL questionnaire, with higher scores indicating better perceived health-related quality of life.

**TABLE 2:** Health-related quality of life of the sample (*N* = 257).

Domain/subscale	Mean	s.d. (%)
Recent physical limitations	81.40	22.25
Symptoms	89.17	17.45
Work-related physical functioning	87.74	23.06
Vitality	52.40	34.01
Section A: Physical health	80.31	15.78
Self-efficacy	62.18	30.35
Challenges to mental health	69.46	33.82
Coping behaviours	74.97	27.80
Treatment literacy	87.20	26.51
Section B: Mental health	72.02	17.19
Perceived social support	79.30	26.12
Financial aspects	82.66	28.47
Relationships	83.96	23.07
Section C: Socioeconomic health	81.49	17.66

**Total**	**77.70**	**12.91**

s.d., standard deviation.

In Table 2, HRQoL and psychosocial well-being were assessed using a 37-item instrument encompassing physical, mental and socioeconomic domains. Overall, the cohort demonstrated a moderate-to-high level of total health status, with a mean total score of 77.70 ± 12.91.

Physical health outcomes were generally high: recent physical limitations scored 81.40 ± 22.25, symptoms 89.17 ± 17.45 and work-related physical functioning 87.74 ± 23.06. The overall physical health section score was 80.31 ± 15.78, indicating that participants experienced relatively few functional limitations despite the presence of chronic conditions. However, vitality was notably lower (52.40 ± 34.01), suggesting reduced energy and potential fatigue within this population.

Mental health domains revealed moderate outcomes. Self-efficacy averaged 62.18 ± 30.35, while challenges to mental health and coping behaviours scored 69.46 ± 33.82 and 74.97 ± 27.80, respectively. Treatment literacy was relatively high (87.20 ± 26.51), reflecting good understanding and engagement with healthcare regimens. The composite mental health score was 72.02 ± 17.19, indicating overall satisfactory psychological well-being with areas for improvement in energy and coping.

Socioeconomic health was favourable, with perceived social support at 79.30 ± 26.12, financial aspects 82.66 ± 28.47 and relationships 83.96 ± 23.07, yielding a composite score of 81.49 ± 17.66. These results suggest participants generally experienced strong social and financial resources, which may buffer the effects of chronic illness.

In summary, while physical and socioeconomic health domains were relatively high, mental health outcomes, particularly vitality and self-efficacy, demonstrated greater variability, highlighting target areas for interventions aimed at improving energy, psychological resilience and coping strategies in this population with multimorbidity.

## Validity and reliability

Internal consistency and test–retest reliability of the STI-HRQoL were high, with Pearson’s *r* = 0.89, Spearman’s rho = 0.88 and an intraclass correlation coefficient (ICC) of 0.94, indicating excellent reliability and stability over time (Smith & Morris-Eyton 2023).

## Discussion

This study demonstrates generally high HRQoL amongst individuals living with hypertension, type 2 diabetes, and/or CVD. Despite the chronic and often debilitating nature of these NCDs, participants maintained strong physical and socioeconomic functioning. These findings underscore the pivotal role of continuous medical care, disease monitoring and access to healthcare resources, particularly in urban and semi-urban populations, in sustaining physical well-being.

Consistent with recent evidence, coordinated care and regular engagement with healthcare services have been linked to improved adherence to therapeutic regimens and better functional outcomes in chronic disease populations (Cheng et al. 2025; Kukulska & Garwacka-Czachor 2024). The results therefore highlight the potential for structured clinical follow-up to mitigate the physical limitations commonly associated with chronic NCDs.

The relatively lower scores observed in the vitality domain warrant further consideration. Vitality, which reflects energy levels and fatigue, is a complex construct influenced by both physical and psychological factors (Ware & Sherbourne 1992). Fatigue among people with chronic NCDs is often multifactorial, arising from metabolic dysregulation, disease-related inflammation, reduced physical activity and disrupted sleep (Kobayashi et al. 2025; Park et al. 2024). This finding indicates that, even when physical functioning is maintained, patients may experience subtle yet impactful limitations in day-to-day energy, which can negatively affect quality of life and engagement in self-care behaviours. While this study did not include direct measures of mental health, it is plausible that psychological variables, particularly depressive symptoms, may have contributed to reduced vitality scores. Depression is well documented to be associated with fatigue, low energy and reduced motivation, all of which are captured within the vitality construct (Bower 2014; Kroenke et al. 2010). Previous research has demonstrated strong associations between depressive symptomatology and diminished HRQoL, particularly in domains related to energy and well-being (Moussavi et al. 2007). In addition to psychological factors, broader determinants such as chronic disease burden, socioeconomic status and physical functioning may also influence vitality. Future research should incorporate validated measures of mental health to better understand these relationships and inform targeted interventions.

Mental health outcomes, while moderate overall, revealed key areas requiring targeted intervention, particularly in relation to self-efficacy. Self-efficacy is a broad construct, generally defined as an individual’s belief in their ability to organise and execute actions required to manage prospective situations (Bandura 1997). Within the context of health research, a distinction is often made between general self-efficacy and domain-specific forms, such as health-related self-efficacy. Within the STI-HRQoL framework, self-efficacy refers specifically to individuals’ perceived ability to manage their health condition and engage in self-care behaviours, rather than a generalised sense of personal competence. Perceived social support reflects the availability of emotional, informational and practical support from others, whereas relationships refer more broadly to the quality and stability of interpersonal connections.

The relatively low self-efficacy scores observed in this study align with evidence that perceived control and confidence are important determinants of self-management and quality of life in chronic disease (Achury-Saldaña et al. 2025; Striberger et al. 2023). Individuals with low self-efficacy are less likely to engage in regular physical activity, adhere to pharmacological treatment or adopt recommended dietary changes, increasing the risk of disease progression and complications.

This suggests that the challenges associated with low self-efficacy are not only behavioural but also cognitive and motivational in nature, including reduced confidence in self-management, maladaptive health beliefs and limited readiness for sustained behaviour change. Psychological interventions such as cognitive behavioural therapy (CBT) and motivational interviewing (MI) are therefore particularly relevant, as they directly target these underlying mechanisms. Cognitive behavioural therapy focuses on restructuring maladaptive beliefs and enhancing coping and problem-solving skills, while MI aims to resolve ambivalence and strengthen intrinsic motivation for behaviour change (Bandura 1997; Beck 2011; Miller & Rollnick 2013). Together, these approaches can enhance self-regulation, improve adherence and support sustained lifestyle modification.

Conversely, high coping behaviours and treatment literacy indicate that participants possess strong adaptive strategies and a solid understanding of their health conditions. These strengths represent a valuable foundation upon which educational and community-based initiatives can build. Programmes such as peer-led education, self-management workshops and group-based interventions have been shown to enhance engagement, reinforce positive health behaviours and strengthen social connectedness, ultimately improving self-confidence and disease outcomes (Holman & Lorig 2004).

The study also revealed strong socioeconomic health outcomes, with participants reporting high levels of relationship quality, financial security and social support. These findings are particularly meaningful in the South African context, where structural inequality and poverty often compromise health outcomes. Social determinants of health, including income, employment, education and social networks, consistently influence both physical and psychological well-being (House, Landis & Umberson 1988; McMaughan, Oloruntoba & Smith 2020; Moscrop et al. 2019). The current results reinforce the protective and buffering effects of social support, suggesting that sustained investment in community health networks and social protection mechanisms may have substantial benefits for HRQoL among populations living with NCDs.

Collectively, these findings align with Engel’s biopsychosocial model of health (1977), which posits that biological, psychological and social factors interact to shape health outcomes. Effective NCD management therefore requires an integrated approach that addresses not only physiological control but also mental health support, behavioural strategies, and social empowerment. This approach is further supported by the World Health Organization’s Integrated People-Centred Health Services framework (WHO 2023), which advocates for holistic, participatory healthcare delivery that places patients at the centre of decision-making and care planning. By adopting such integrative models, healthcare systems can better support individuals in achieving optimal HRQoL despite the burdens of chronic disease.

### Limitations and recommendations

The findings of this study highlight the need for integrated, multidimensional strategies to strengthen chronic disease management. Psychosocial support should be incorporated into routine care, with the use of brief counselling, MI, CBT and fatigue-management programmes to address reduced vitality and self-efficacy. Establishing clear referral pathways to mental health professionals is similarly important. Multidisciplinary, team-based care involving nurses, biokineticists, dietitians, psychologists and social workers is recommended to meet the diverse needs of patients and improve self-management capacity. Lifestyle-focused interventions should also be prioritised, including community-based physical activity programmes, sleep hygiene and stress-reduction education and nutritional counselling to support energy levels and metabolic control. While treatment literacy was relatively high, ongoing patient education remains essential for promoting adherence and disease understanding. Digital health technologies such as mobile applications, short message service (SMS) reminders and telehealth follow-ups offer additional opportunities to enhance accessibility, facilitate monitoring and reinforce behaviour change. Strengthening social support through community health workers and peer-support groups may further bolster resilience, coping and adherence. Finally, future research should include longitudinal studies to monitor HRQoL trajectories over time, qualitative exploration of lived experiences to better understand the factors driving low vitality and self-efficacy and examination of structural barriers that influence self-management among individuals living with multimorbidity. Given the relatively low vitality scores observed, further research is warranted to better understand the underlying factors contributing to reduced energy levels and fatigue within this population. In particular, future studies should aim to identify the relative contribution of psychological factors such as depression and stress, physical health status and socio-economic conditions to vitality outcomes. Such insights would be critical in informing the development of targeted, context-specific interventions aimed at improving vitality and overall HRQoL.

Although this study provides important insight into HRQoL within the study population, the absence of more detailed comparative and predictive analyses should be acknowledged. The application of multivariable regression analysis and subgroup comparisons (e.g. employment status or healthcare sector) may have provided further insight into the determinants of HRQoL. However, the study was not originally designed to support such analyses. Future research should incorporate these approaches to strengthen the understanding of HRQoL and its predictors.

This study has several notable strengths. It employed a comprehensive and validated 37-item HRQoL instrument that captures physical, mental and socioeconomic dimensions, offering a holistic understanding of patient well-being. The inclusion of participants from both public and private healthcare facilities enhanced representativeness and allowed for meaningful comparisons across socioeconomic contexts. The sample was demographically and clinically diverse, spanning a wide age range (21–88 years), multiple ethnic groups and varied employment and educational backgrounds, thereby strengthening the generalisability of the findings.

Recruitment during routine clinical consultations further reduced selection bias and enhanced ecological validity by reflecting real-world patient experiences within chronic disease management pathways. Moreover, the high reliability of the HRQoL instrument (Pearson *r* = 0.89, Spearman rho = 0.88, ICC = 0.94) provides confidence in the robustness and accuracy of the reported outcomes.

Despite these strengths, several limitations should be acknowledged. The cross-sectional design restricts the ability to infer causal relationships between HRQoL domains and patient characteristics. The study was conducted within urban Johannesburg healthcare settings, which may limit the applicability of findings to rural or peri-urban populations with differing healthcare access or resource constraints. As data were self-reported, results may be influenced by recall and social desirability biases, as well as subjective interpretation of HRQoL items. There is also a possibility of selection bias, as individuals who agreed to participate or who completed the online version, may differ systematically from those who declined, particularly regarding motivation, health literacy or digital access. Finally, several potentially important confounders, including disease severity, medication regimen, duration of illness and detailed comorbidity profiles, were not measured and may have influenced HRQoL outcomes.

## Conclusion

This study demonstrates that individuals living with hypertension, type 2 diabetes and/or CVD maintain generally high health-related quality of life, particularly in physical and socioeconomic domains, despite the chronic nature of their conditions. Notably, mental health outcomes, particularly vitality and self-efficacy, remain areas of vulnerability, highlighting the complex interplay between physiological, psychological and social factors in shaping overall well-being. These findings underscore the importance of a holistic, patient-centred approach to NCD management that extends beyond clinical control of disease to address psychosocial and lifestyle dimensions.

From a policy and practice perspective, these findings suggest that strengthening psychosocial interventions may yield substantial benefits for chronic disease management. Interdisciplinary teams that include biokineticists, psychologists and social workers could provide comprehensive care addressing both physical and mental needs.

Additionally, digital health tools such as telemedicine and mobile health applications can support ongoing education, monitoring and motivation, particularly in resource-constrained settings.

Future research should focus on longitudinal tracking of HRQoL changes over time to better understand the trajectory of physical and psychological adaptation in chronic illness. Moreover, qualitative exploration of patients’ lived experiences could illuminate contextual factors influencing adherence behaviours, motivation and coping strategies within diverse socioeconomic and cultural environments. Such insights could inform tailored, context-sensitive interventions aimed at enhancing patient engagement and sustaining long-term improvements in HRQoL.
